# Antibiotic prescriptions in Italian hospitalised children after serial point prevalence surveys (or pointless prevalence surveys): has anything actually changed over the years?

**DOI:** 10.1186/s13052-019-0722-y

**Published:** 2019-10-17

**Authors:** Chiara Tersigni, Carlotta Montagnani, Patrizia D’Argenio, Marzia Duse, Susanna Esposito, Yingfen Hsia, Mike Sharland, Luisa Galli

**Affiliations:** 1 Institute for Infection and Immunity, Paediatric Infectious Disease Research Group, St. George’s, University of London, London, England; 2 Post graduate school of Paediatrics, University of Florence, Florence, Italy; 3 Department of Health Sciences, University of Florence, Anna Meyer Children’s University Hospital, Viale Pieraccini 24, 50139 Florence, Italy; 4 Unit of Immune and Infectious Diseases, Academic Department of Pediatrics, Children’s Hospital Bambino Gesù, Rome, Italy; 5 Department of Pediatrics, La Sapienza University of Rome, Rome, Italy; 6 Pediatric Clinic, Department of Surgical and Biomedical Sciences, University of Perugia, Perugia, Italy

**Keywords:** Antimicrobial stewardship, Children, Antimicrobials, Point prevalence surveys

## Abstract

**Background:**

Point prevalence surveys have been used in several studies to provide immediate and easily comparable information about antibiotic use and showed that about one third of hospitalised children had on ongoing antimicrobial prescription during their hospital admission. The aim of this study, as part of the Global Antimicrobial Resistance, Prescribing and Efficacy in Neonates and Children project, is to describe antimicrobial prescriptions among hospitalised children in four tertiary care hospitals in Italy to show if something has changed over the years.

**Methods:**

Four tertiary care Italian’s hospitals joined three Point Prevalence Surveys (PPSs) in three different period of the year. All children under 18 years of age with an ongoing antimicrobial prescription, admitted on the participating wards at 8 o’clock in the morning of the selecting day were enrolled.

**Results:**

A total of 1412 patients (475 neonates and 937 children) were admitted in the days of three PPSs. Overall, among the total admitted patients, 565 patients (40%) had an ongoing antimicrobial prescription in the days of the survey A total of 718 antibiotics were administered in the 485 admitted children and 133 in neonates. The most common indications for antibiotic therapy in children was Lower respiratory tract infections (244/718, 34%), while in neonates were prophylaxis for medical problems (35/133, 26.3%), newborn prophylaxis for newborn risk factors (29/133, 21.8%) and prophylaxis for surgical disease (15/133, 11.3%).

**Conclusions:**

Based on our results, it appears that nothing has changed since the last PPS and that the quality improved targets, underlyined in previous studies, are always the same. Serial PPSs can be part of AMS strategies but they are not sufficient alone to produce changes in clinical practice.

## Background

According to the World Health Organization (WHO)‘s statements, antimicrobial resistance (AMR) is one of the major contemporary threats to public health, both for epidemiological and economic reasons [1]. Several studies have demonstrated that AMR development is related to broad spectrum antimicrobial use and that the optimization of AM prescriptions is a key point of antimicrobial stewardship programs (ASPs) [2]. Repeated Point Prevalence Surveys (PPSs), conducted in several European and non-European countries, demonstrate high broad spectrum antibiotic use in hospitals [3–6].

The aim of this study, as part of the Global Antimicrobial Resistance, Prescribing and Efficacy in Neonates and Children (GARPEC) project, was to compare antimicrobial prescriptions among hospitalised children in four tertiary care hospitals in Italy using the same methodology from previous pediatric PPS’s, to investigate if previous PPS studies had led to any change in clinical prescribing practice.

## Methods

The present study is part of the GARPEC, the global study that followed the European Antibiotic Resistance and Prescribing in European Children project (ARPEC) [3–6]. Three PPSs were conducted: the first one from May to June 2016, the second one from August to September 2016, and the last one from November to December 2016. Four tertiary care university Italian hospitals participated three PPSs. The methodology and data collection has been described elsewhere [3–8]. A comparison between the results of the present study and two other studies conducted in Italian institutions, with the same methodology, is reported in Table 1 [4, 5].

**Table 1:** Comparison between the present study and previous PPS studies (4,5)

	Ciofi Degli Atti et al. (4)	De Luca et al. (5)	Present study
Overall			
Year	June 2007	October–December 2012	February – December 2016
Number of PPSs	1	1	3
Participating hospitals	1	7	4
Percentage of patients with an ongoing AMs** prescription	43.9%	38.9%	40%
Mean prescription/treated patient	1.4	1.56	1.32
Combination therapy/treated patients	43.8%	41.3%	41.9%
AMs prescribed empirically	51%	–	72.6%
AMs use in Neonates			
Percentage of neonates receiving AMs	-*	17.3%	16.4%
AMs used for prophylaxis	–	62.8%	59.4%
AMs used for treatment	–	37.2%	40.6%
Most common reason for treatment	–	Sepsis	Sepsis
Top two prescribed AMs	–	Penicillins	Aminoglycosides
		Aminoglycosides	Ampicillin
AMs use in Children			
Percentage of children receiving AMs	–	47%	51.8%
AMs used for prophylaxis	–	35.5%	21.3%
AMs used for treatment	–	64.4%	78.7%
Most common reason for treatment	–	LRTI	LRTI
Top two prescribed AMs	Third generation cephalosporins	Third generation cephalosporins	Third generation cephalosporins
	Penicilins plus enzyme inhibitor	Penicilins plus enzyme inhibitor	Penicilins plus enzyme inhibitor

Abbreviations: PPSs: point prevalence surveys; AMs: antimicrobials; LRTI: low respiratory tract infection

* Lacking of analysis disaggregated by age

### Statistical analysis

Statistical analysis was performed using SPSS (version 25.0, SPSS Inc., Chicago, IL). Metric data were tested for normal distribution. Results were expressed as mean (standard deviation, SD) or median (interquartile range, IR) as appropriate. Unpaired *t* test and Mann-Whitney tests were used to compare variables between groups. The χ-square test and Fisher test were performed when appropriate.

## Results

A total of 1412 patients (475 neonates and 937 children) were admitted on the days of three PPSs were carried out. Overall 565 patients (40%; 565/1412) received at least one antibiotic prescription on the days of the survey, with 51.8% (485/937) and 16.4% (80/475) in children and neonates, respectively. Of these, 34.6% (114/329) in the first PPS, 39.8% (220/552) in the second PPS, and 47.3% (231/531) in the third PPS; *p* < 0.005).

### Antibiotics prescribed in children

A total of 718 antibiotics (an average of 1.48 antibiotic prescriptions per children) were administered in children (aged > 1 months) with no significant differences between three PPSs (*p* = 0.199). Excluding antibiotics prescribed for prophylaxis, a statistically significantly higher proportion of antibiotics were prescribed in the second and in the third PPS (*p* < 0.001). About 41.9% of children received a combination of two or more antibiotics. The two most common indications for antibiotic prescribing were lower respiratory tract infection (LRTI) (34%) and prophylaxis for surgical disease and for medical problems (14.3 and 7.4% respectively). Overall, the most commonly prescribed antibiotics were the third generation cephalosporins (26.3%), penicillin with enzyme inhibitor (18.1%), and aminoglycosides (12.7%). The parenteral route of administration was used in 590/718 cases (82.2%). Almost half of patients received antibiotics reported as community acquired infections (CAI) (50.8%; 365/718 patients), and only 24.9% (179/718 patients) as hospital acquired infections (HAI), and 22.1% (159/718 patients) as prophylaxis. In the cases of antibiotics administered for treatment (560/718, 78%), only 27.4% (154/560) of antibiotics were prescribed targeted treatment with pathogen and antimicrobial susceptibility test confirmed.

### Antibiotics prescribed in neonates

Overall, 16% (80/475) neonates received at least one antibiotic in the days when PPSs were carried out. Overall, 80 neonates received at least one antibiotics in the days of three PPSs were carried out. Neonates with reported underlying conditions were more likely to receive antibiotic for treatment compare with those without underlying condition during the surveys (77.5% vs 22.5%, *p* < 0.001). Prematurity was the most common reported underlying conditions in our study population (32.5%; 25/80 patients). Regarding antibiotic prescription, a total of 133 antibiotics were administered (an average of 1.66 antibiotic prescriptions per neonate). The most commonly prescribed antibiotic, alone or in combination, was ampicillin or ampicillin and enzyme inhibitor (27.8%) followed by gentamycin (18.8%).

When the indication for antibiotic use was reported (127/133), antibiotics were administered for treating HAIs in 21.3% (27/127), for CAIs in the 15.7% (20/127) and for prophylaxis in 63% (80/127). The parenteral route of administration was preferred in 128/133 cases (96.2%). The most common reasons for antibiotic treatment in neonates were prophylaxis for medical problems (26.3%), newborn prophylaxis for neonatal risk factors (21.8%), prophylaxis for surgical disease (11.3%), sepsis (11.3%), treatment for surgical disease (10.5%) and LRTI (9%). The two most commonly prescribed antibiotics for medical prophylaxis were (alone or in combination) gentamycin (9/35, 25.7%) and ampicillin (8/35, 22.8%).

### Comparison with previous studies

Two other studies, using the PPSs methodology have been published in the literature in 2007 and in 2012, respectively (4,5). The percentage of patients with an antibiotic prescription, the number of antibiotics/treated patients, the percentage of antibiotics prescribed empirically and the characteristics of antibiotic use in children and neonates are reported in Table 1.

## Discussion

The present study provides an estimate of antibiotic use in children and neonates in four tertiary care Italian hospitals, in three different seasons, using a standardised PPS methodology. About 40 % of admitted patients received at least one antibiotic during their hospital stay; however, when new-borns are excluded, this percentage increases to more than 50%. These data are concerningly, very similar to the previous PPS studies conducted in 2007 and in 2012 in Italian tertiary care hospitals (Table 1) [3–9]. The three PPSs reported in the present study, conducted about nine and seven years after the previous ones, respectively, did not reveal any reduction in overall AMs use. A number of specific quality improvement targets, identified as key points in ASPs, have been developed [1–3]. The most commonly prescribed remained antibiotics were third generation cephalosporins (prescribed as first choice in LRTIs, skin and soft tissue infections and joint and bone infections) and penicillin with enzyme inhibitor and aminoglycosides. There remained an extensive use of antibiotics for prophylaxis, both for medical and surgical reasons, which is not supported by available guidelines [10]. There was very high rates of prescribing for prophylaxis in neonates (60.1%). However, even though the neonatal population with risk factors (i.e. prematury) can be considered particulary vulnerable since invasive procedures are often necessary and because of the presence of an immature immune system, the huge use of antibiotics for prophylaxis cannot be considered evidence-based [8, 9, 11].

## Conclusions

PPS is an easy method to perform and monitor antibiotic use, with the main aim of raising awareness of the rationale of antibiotic prescriptions in the paediatric population. However, our study showed that PPSs by themselves are not sufficient to produce any changes in the clinical practice if they are not sustained by ASPs. Based on the present study, it appears that nothing has changed since the last PPSs [4, 5] and that the quality improvement targets, underlined in previous studies, are always the same. More attention should also be focused on whether this has been noted in other studies. It is unclear why there has been limited evidence of improvement in the quality of prescribing. There is very little published evidence of the optimal design of stewardship interventions in hospitalised children [12, 13]. The data presented here suggests that PPS need to be more specifically combined with simple, cheap and easily implemented stewardship interventions.

## Data Availability

The datasets used and/or analysed during the current study are available from the corresponding author on reasonable request.
